# Macro- and micro-anatomical investigation of the oropharyngeal roof of landform greek tortoise (*Testudo graeca graeca)* and semi-aquatic red-eared slider turtle (*Trachemys scripta elegans)*

**DOI:** 10.1186/s12917-024-04157-x

**Published:** 2024-07-11

**Authors:** Mohamed A.M. Alsafy, Nermin K.A. El-Sharnobey, Samir A.A. El-Gendy, Mohamed A. Abumandour, Samar M. Ez Elarab, Basma G. Hanafy

**Affiliations:** 1 Anatomy and Embryology Department, Faculty of Veterinary Medicine, Alexandria University, Abis 10th, P.O. 21944, Alexandria, Egypt; 2 Histology and Cytology Department, Faculty of Veterinary Medicine, Alexandria University, Abis 10th, P.O. 21944, Alexandria, Egypt

**Keywords:** Turtles and tortoises, Oropharyngeal roof, Gross morphology, Light microscopy, Scanning electron microscopy

## Abstract

The present investigation examined the oropharyngeal roof of two turtles having different feeding behaviors: the landform Greek tortoise (*Testudo graeca graeca*) primarily herbivores and the semi-aquatic red-eared slider turtle (*Trachemys scripta elegans*) lives in freshwater that opportunistic omnivorous grossly and by scanning and light microscopes. Grossly, the Greek tortoise had a V-shaped roof consisting of the upper rhamphotheca, peri-palatine region, upper alveolar ridge, peripheral palatine ridge, median palatine ridge, vomer, choanae, caudal palatine part, and pharynx. At the same time, the red-eared slider had a semilunar roof consisting of upper rhamphotheca, two peripheral palatine ridges, core of palatine ridges, upper alveolar band, vomer, choanae, caudal palatine part, and pharynx. SEM revealed that the red-eared slider roof appeared more straightforward. The upper rhamphotheca is sharp, with a median premaxillary notch in the red-eared slider that gives a powerful bite for cutting to compensate absence of the teeth. Additionally, the red-eared slider’s upper alveolar band is interrupted by a single upper alveolar ridge that appears spiky, pointed, and longer as it needs powerful chewing of prey and there are two types of teeth-like projections at its peri-palatine area for food-crushing and chewing. The Greek tortoise palatine region had numerous ridges and folds to provide roughness for food processing. Greek tortoises had small-sized choanae with two choanal folds to minimize choanal openings when eating dusty grasses. Histologically, Greek tortoise palate was rostrally thicker and more keratinized than caudally, and the caudal palatine region was characterized by a single pair of circumvallate-like papilla with multiple mucous openings and secretions, while red-eared slider palate was slightly keratinized at the peri-choanal region, and the rest of the palate was non-keratinized with few mucous openings. The current investigation found various structural oropharyngeal roof adaptations to feeding behavior in the omnivore red-eared slide compared to the herbivorous Greek turtle.

## Introduction

Turtles are a type of reptile that belongs to the Suborder Cryptodira and Superfamily Testudinoidea, which includes four families: Testudinidae (tortoises), Emydidae, Geoemydidae, and Platysternidae; these four families contain 320 known species [1, 2]. Turtles and tortoises (chelonians) have played an essential role in ecosystems around the world since approximately 220 million years ago, inhibiting a variety of fully terrestrial, freshwater, and marine ecosystems [3]. Turtles were recorded in Egypt for the first time Anderson 1898 through the book Zoology of Egypt [4].

*Testudo graeca graeca*, also known as Greek tortoises or spur-thighed tortoises, live primarily in North Africa and Southern Europe. However, they also have small and isolated populations in Spain and some Mediterranean islands [5]. Although they do not typically live in deserts because they prefer grasslands, they can tolerate dry and hot environments in deserts [6]. They are primarily herbivores that consume vegetables, grasses, various plants, and fruits [7].

*Trachemys scripta elegans*, also known as red-eared sliders, pond sliders, or red-eared terrapins [8], is a native indigenous species of the United States, particularly in the southern sector [9]. However, as a result of their release as unwanted pets, they began to crawl to other ranges outside their natural range and were introduced to some places and countries in Europe, Asia, Africa, and Australia [10]. *Trachemys scripta* is an opportunistic omnivorous reptile that consumes a variety of invertebrates (shrimps, shellfish, crabs, snails, insects), vertebrates (fish, rodents, frogs, lizards, birds, snakes), and aquatic plants such as ferns, algae, and seed plants [9]. *Trachemys scripta* species consume a significantly different proportion of animal and plant materials, with sliders preferring fleshy products over plant ones [11].

The oral cavity of non-avian reptiles, such as turtles, serves several functions, including thermoregulation, defense, respiration, mating behavior, feeding, and swallowing [12, 13]. However, the primary function of the oropharynx has been linked to food processing and swallowing, so the size of the oropharyngeal cavity varies depending on the feeding mechanism. Aquatic turtles have large oral cavities and large volume expansion to allow for suction feeding underwater using negative pressure [14], whereas most terrestrial tortoises have a small oral cavity and rely on jaw prehension. A functionally movable tongue aids in lingual food prehension and intraoral transport [15].

Turtles have no teeth but a keratinized beak (rhamphotheca) that covers their bony jaws. These well-developed, horny, sharp beaks act as functional alternatives to teeth for tearing and crushing food [16]. Most turtles’ palates resemble primitive vertebrates like fish and amphibians [17]. Turtles’ palates are also bounded laterally and rostrally by the dorsal horny beak, as well as caudally by the pharynx. All reptiles have incomplete palates, except crocodilians, which have a fully developed secondary palate [17]. Vomer expands to divide and separate the left and right choanal openings [18]. Vomer clefts appeared in the Pancake tortoise (*Malacochersus tornieri*), while the eastern long-necked turtle (*Chelodina longicollis*) had the widest vomer [19]. Keratinization of the oral mucosa is common in land tortoises and purely aquatic turtles, providing protection from abrasion or dehydration caused by poor and dry environmental conditions, whereas semiaquatic turtles have less or no keratinization [20]. The morphologic and histologic descriptions of turtles’ buccopharyngeal cavities differ significantly, which may be attributed to differences in feeding habits, processed food, body format, and, most importantly, the diverse environmental conditions in which they live [15].

The current study focused on the anatomical and histological details of the oropharyngeal roof of Egypt’s most famous pet turtles and tortoises, the Greek tortoise (*Testudo graeca graeca*) and red-eared slider *(Trachemys scripta elegans)*, and how they relate to the morphological structure and oral cavity of turtles with different feeding habits.

## Materials and methods

### Tortoises and turtles

Twelve adult turtles of two different species (8 males and 4 females) were used in the current study: six adult Greek tortoise *(Testudo graeca graeca)* with carapace lengths ranging from 20.7 cm to 22.1 cm and weights ranging from 1.18 kg to 1.28 kg and six adult red-eared slider *(Trachemys scripta elegans)* with carapace lengths ranging from 23.4 cm to 25.1 cm and weights ranging from 1.41 kg to 1.73 kg.

The examined turtles were obtained from a local pet shop in Alexandria, Egypt. All twelve animals were brought to the anatomy laboratory in travel pet cages. Then, they were kept for (3 days) a while to ensure they were healthy and free of any oral abnormalities or injuries. So, the Greek tortoises were kept in a small yard with sand and stones and fed cucumbers, carrots, and lettuce. In contrast, the red-eared sliders were kept in an aquarium with small and large stones and provided a variety of commercial pellets, small fish such as sardines, and vegetables.

Six Greek tortoises were anesthetized by ketamine (50 mg/kg) in combination with xylazine (2 mg/kg) intramuscularly in the pectoral muscle. At the same time, the red-eared sliders needed a high dose of ketamine (60 mg/kg) with (2 mg/kg) xylazine intramuscular [21]. After deep narcosis, all animals were decapitated.

### Gross morphology

Two heads of Greek tortoises and two heads of red-eared sliders were divided entirely horizontally through the mouth until the esophagus into two parts: the upper jaw showing the roof of the oral cavity and the lower jaw showing the oral cavity floor [22]. Then, for gross morphological imaging of the oropharyngeal roof, all roofs were photographed using a Canon EOS 2001 digital camera.

### Light microscopy (LM)

Two heads of Greek tortoises and two heads of red-eared sliders were used. Fresh samples of 0.5 cm^3^ from the palate, choanae, and pharynx of both the red-eared slider and the Greek tortoise were fixed in 10% phosphate-buffered formaldehyde. Then, they were processed for paraffin sectioning. Serial Sect. (4 μm) were prepared by microtome and stained with Mayer’s hematoxylin and eosin stain (H&E) for general studies [23, 24]. Masson’s trichrome for collagen fiber and muscle fiber [25] and Periodic Acid Schiff (PAS) technique for mucopolysaccharides and neutral mucin [26]. The slides were examined with an Optical Italian microscope.

### Scanning electron microscopy (SEM)

Two heads of Greek tortoises and two heads of red-eared sliders were used. The oropharyngeal roofs were fixed in a buffer solution of 2% formaldehyde, 1.25% glutaraldehyde, and 0.1 M sodium cacodylate at pH 7.2 and 4 °C. After fixation, the samples were washed in 0.1 M sodium cacodylate containing 5% sucrose and finally dehydrated in increasing grades of ethanol (15 min each in 50%, 70%, 80%, 90%, 95%, and 100% ethanol) [27]. The samples were dried in carbon dioxide, attached to stubs with colloidal carbon, and coated with gold-palladium in a sputtering device. Specimens were examined and photographed with a JEOL JSM-IT200 scanning electron microscope at 15 kV at the electron microscope unit, Faculty of Science, Alexandria University [28].

## Results

### Gross morphological features of the oropharyngeal roof

**Fig. 1:**
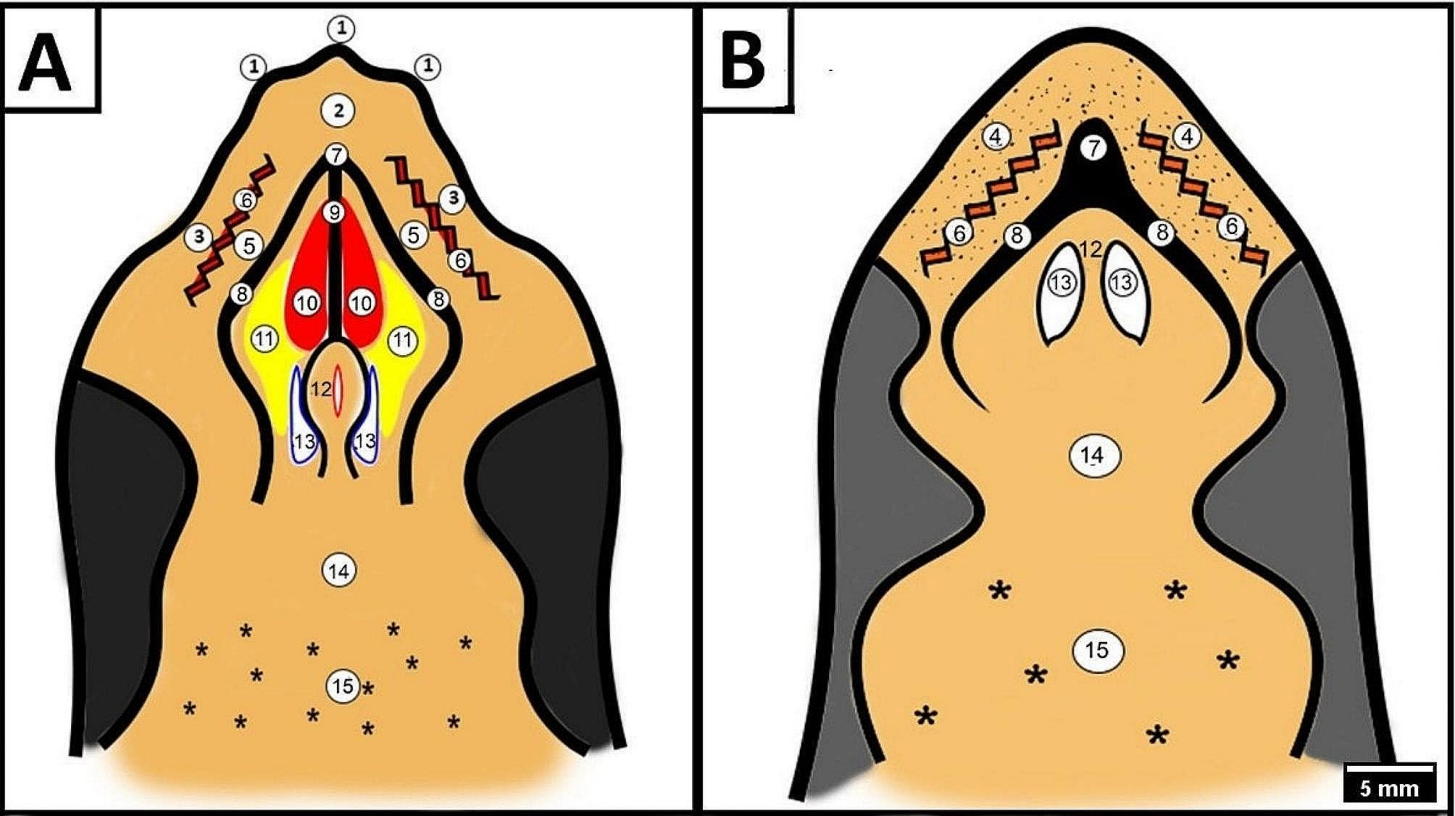
Schematic drawing shows the roof of the oropharyngeal cavity of Greek tortoise (view A) and red-eared slider (view B); Explined the following; the tomium (1), peri-palatine area (2), upper alveolo-beakal groove (3), upper alveolar band (4), upper alveolo-palatine groove (5) upper alveolar ridge (6), core of palatine ridge (7), peripheral palatine ridge (8), median palatine ridge (9), palatine fold (10), choanal fold (11), vomer (12), choanal opening (13), caudal palatine region (14), pharynx (15) and pharyngeal salivary glands (black asterisk)

In both species, the roof of the oropharyngeal cavity was larger than the floor; the lateral surface of the lower rhamphotheca rested on the inner surface of the upper rhamphotheca during their mouth closure, and it was demarcated rostro-laterally by the upper rhamphotheca and caudally by the pharynx (Fig. 1A, B).

**Fig. 2:**
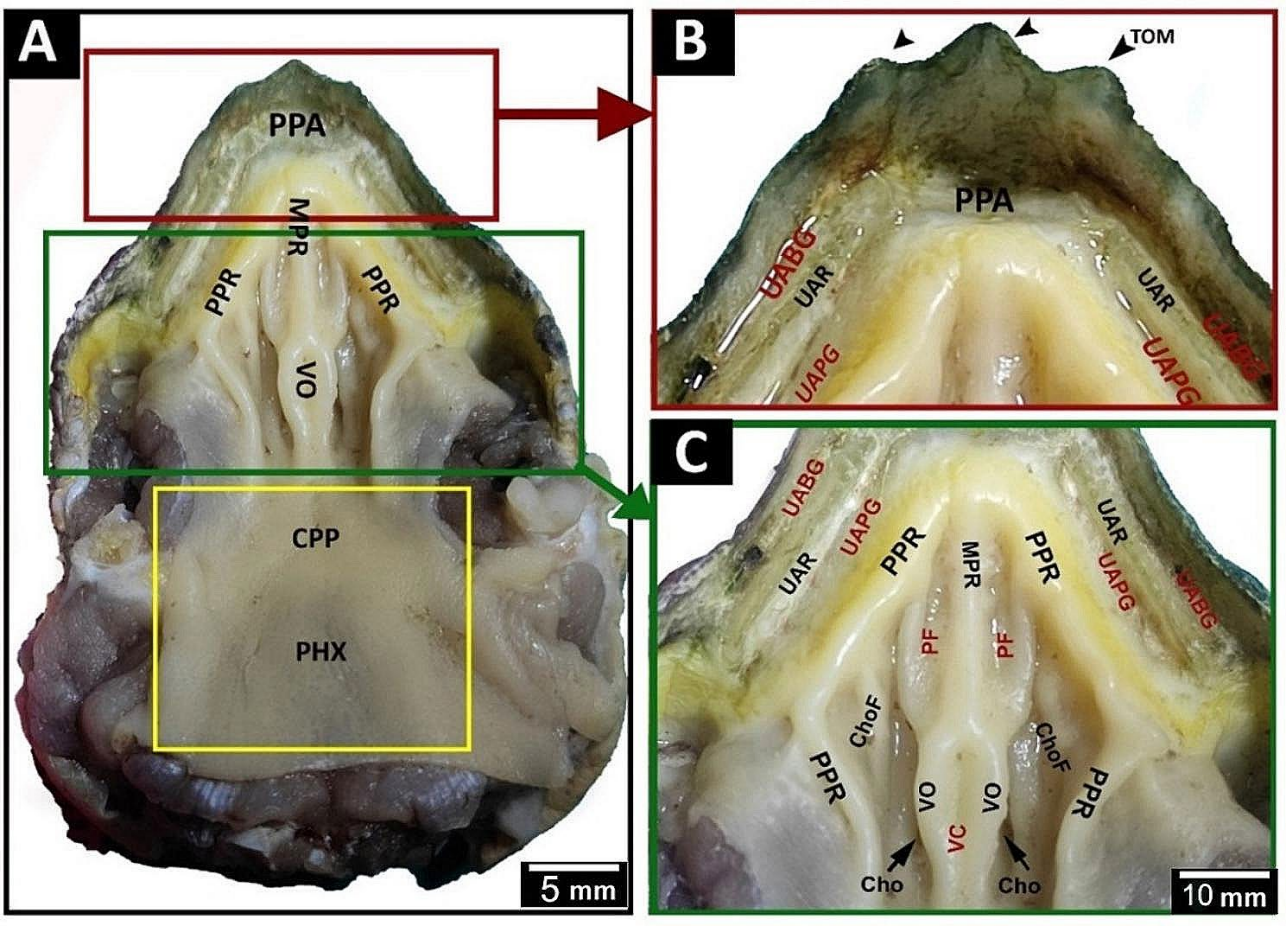
gross images show morphological features of the Greek tortoise oropharyngeal cavity roof. View (**A**) shows a dorsal view of the whole roof. View (**B**) indicates the rostral palatine region. View (**C**) shows the middle palatine region. Prei-palatine area (PPA), peripheral palatine ridge (PPR), median palatine ridge (MPR), vomer (VO), caudal palatine part (CPP), pharynx (PHX), tomium (TOM), upper alveolar ridge (UAR), upper alveolo-beakal groove (UABG), upper alveolo-palatine groove (UAPG), palatine fold (PF), vomer cleft (VC), choanae (Cho), choanal fold (ChoF)

**Fig. 3:**
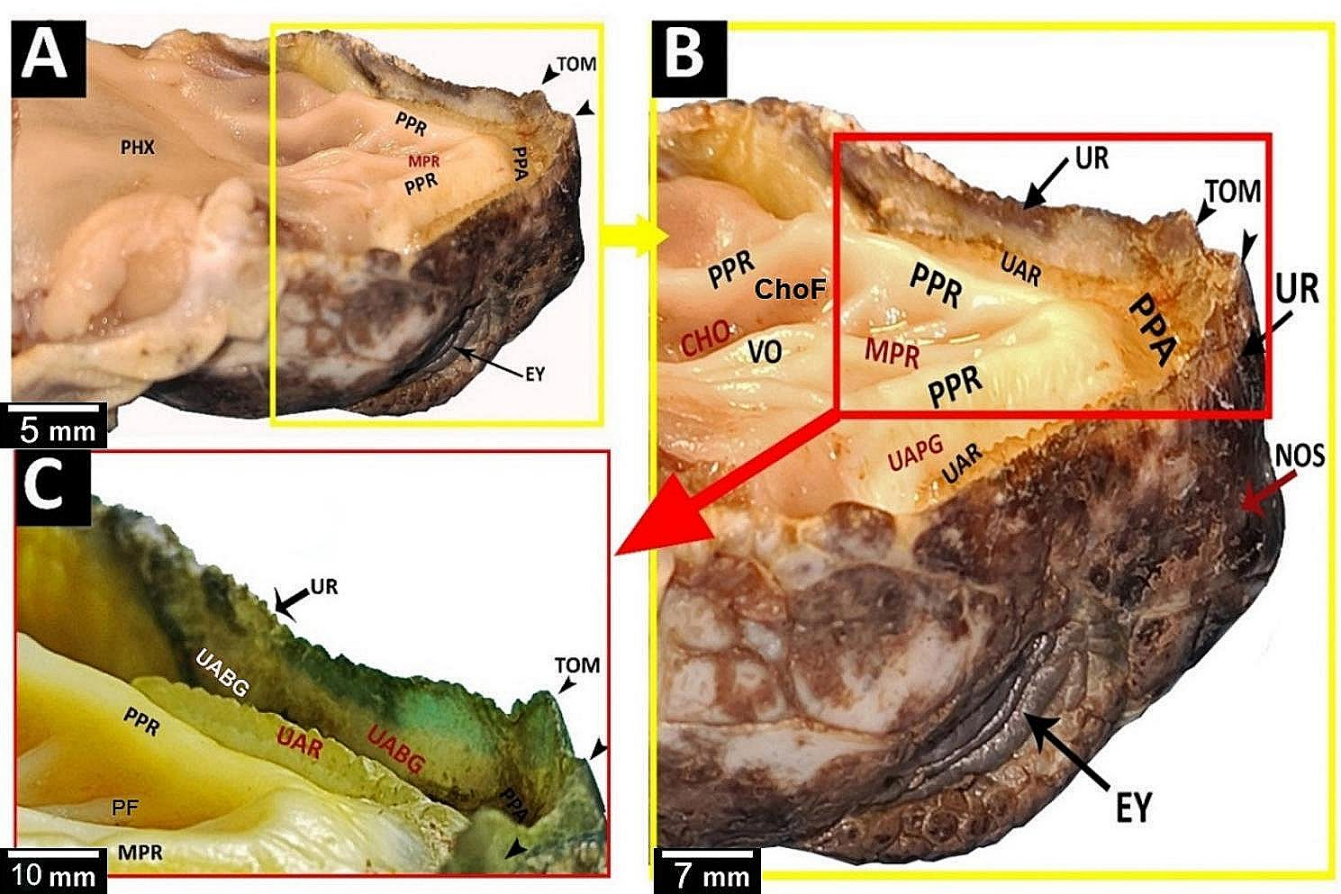
An image shows a dorsolateral view of the Greek tortoise oropharyngeal cavity roof in view (**A**), Magnification of the area marked by the yellow rectangle in view (**B**), and Magnification of the area marked by the red rectangle in view (**C**). Tomium (TOM), Prei-palatine area (PPA), peripheral palatine ridge (PPR), median palatine ridge (MPR), vomer (VO), pharynx (PHX), upper alveolar ridge (UAR), upper alveolo-beakal groove (UABG), upper rhamphotheca (UR), upper alveolo-palatine groove (UAPG), palatine fold (PF), vomer cleft (VC), choanae (Cho), choanal fold (ChoF) eye (EY), nostrils (NOS)

Greek tortoise had a V-shaped oropharyngeal roof consisting of the upper rhamphotheca, pre-palatine area, upper alveolar ridge, two peripheral palatine ridges, median palatine ridge, vomer, choanae, caudal palatine part and pharynx (Fig. 2A, B, C). Rostrally, the oropharyngeal cavity roof of the Greek tortoise had an upper rhamphotheca with serrated edges called tomium with three prominent projections (Fig. 3A, B, C). V-shaped space appeared caudally to the upper rhamphotheca; it encircled the palate, appeared rostrally as a transverse pre-palatine area, and was divided laterally by serrated upper alveolar ridges into outer upper alveolo-beakal groove and inner upper alveolo-palatine groove (Figs. 2A, B and C and 3A and B). The middle part of the oropharyngeal cavity roof of Greek tortoise had two peripheral palatine ridges and a single median palatine ridge that continued caudally toward caudal palatine part and pharynx as a vomer separated the right and left choanal openings (Fig. 3B). The surface of the vomer showed a vomer cleft (Fig. 2C). Additionally, the middle palatine region had two palatine folds and two choanal folds (Figs. 2C and 3C). Each palatine fold separated the median palatine ridge from each peripheral palatine ridge (Figs. 2C and 3B and C). The choanal folds were partially covered with the choanal openings that appeared as narrow oval openings (Figs. 2C and 3B). The caudal palatopharyngeal region showed no gross characteristics (Fig. 2A).

**Fig. 4:**
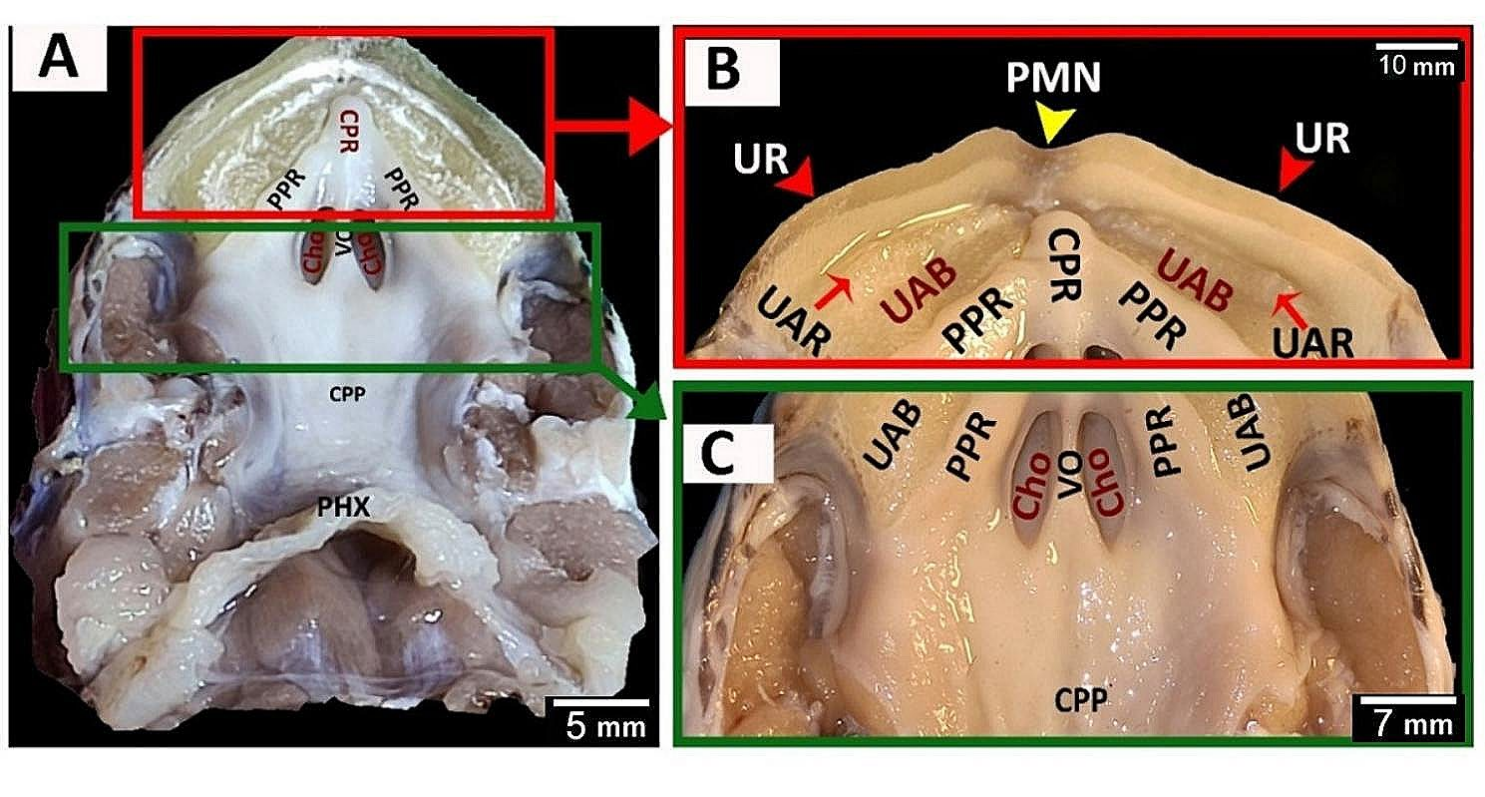
An image shows gross morphological features of the **red-eared slider** oropharyngeal roof. View (**A**) shows a dorsal view of the whole roof. View (**B**) shows a magnification of the area marked by the red rectangle. View (**C**) shows a magnification of the area marked by the green rectangle. Upper rhamphotheca (UR), premaxillary notch (PMN), upper alveolar ridge (UAR), upper alveolar band (UAB), peripheral palatine ridge (PPR), core of palatine ridge (CPR), vomer (VO), choanae (Cho), pharynx (PHX), caudal palatine part (CPP)

**Fig. 5:**
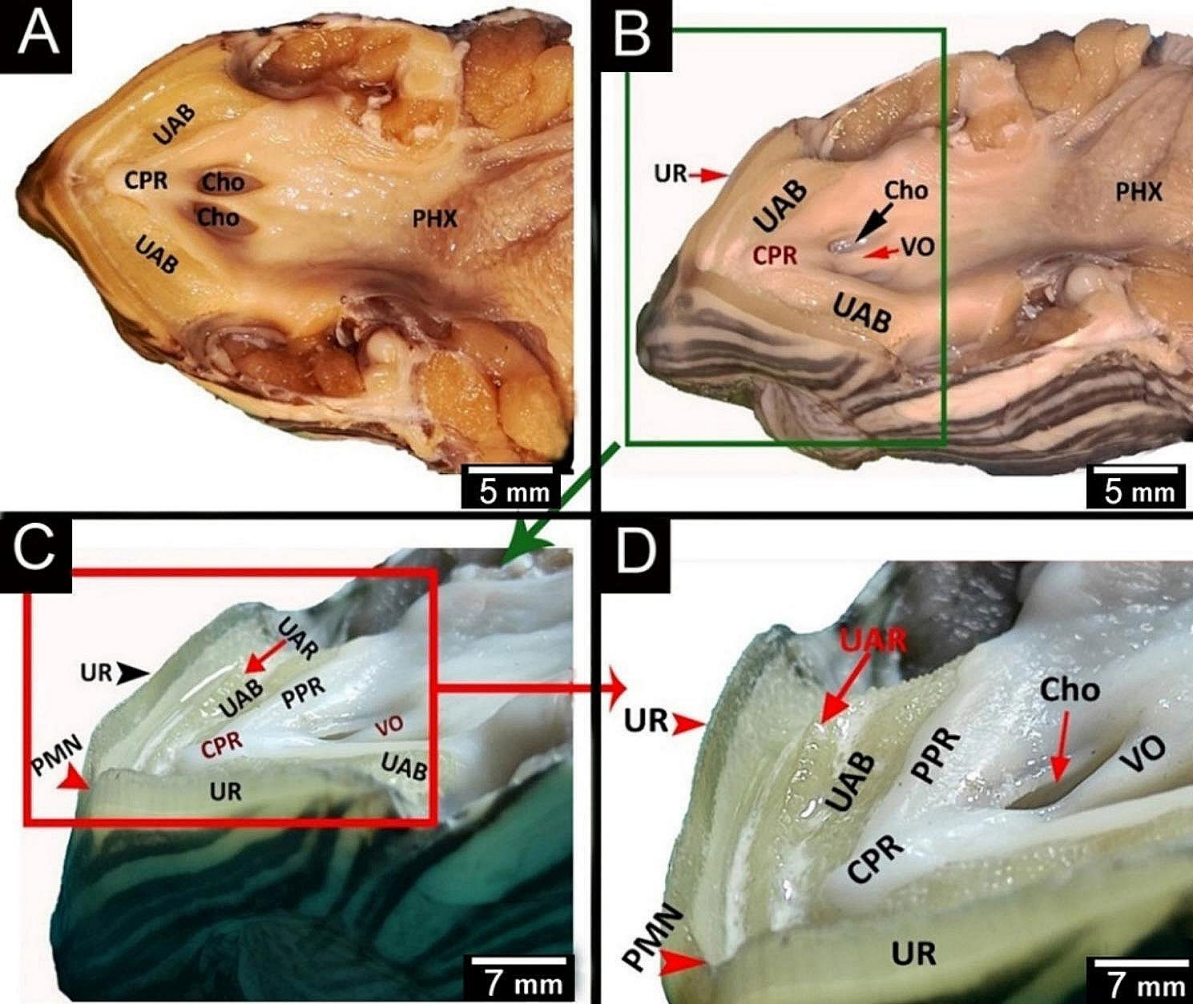
An image shows dorsolateral views of the red-eared slider oropharyngeal cavity roof in views (**A** & **B**), Magnification of the area marked by the green rectangle in view (**C**), and Magnification of the area marked by the red rectangle in view (**D**). Upper rhamphotheca (UR), premaxillary notch (PMN), upper alveolar ridge (UAR), upper alveolar band (UAB), peripheral palatine ridge (PPR), core of palatine ridge (CPR), vomer (VO), choanae (Cho), pharynx (PHX)

The red-eared slider had a semilunar-shaped oropharyngeal roof consisting of upper rhamphotheca, two peripheral palatine ridges, core of palatine ridges, upper alveolar band, vomer, choanae, caudal palatine part and pharynx (Fig. 4A). Rostrally, the oropharyngeal roof of red-eared slider had an upper rhamphotheca, with sharp cutting edge and a median premaxillary notch and rostral parts of two upper alveolar bands, two peripheral palatine ridges and a single core of palatine ridge (Figs. 4A and B and 5B, C and D). The middle part of the oropharyngeal roof of the red-eared slider had a vomer, two elliptical choanal openings and caudal regions of two peripheral palatine ridges, two upper alveolar bands, and a single core of palatine ridges (Figs. 4C and 5A, B and D). The caudal palatopharyngeal region of the red-eared slider did not show any gross characteristics (Fig. 4A, C).

### SEM characterizations of the oropharyngeal roof of Greek tortoise

**Fig. 6:**
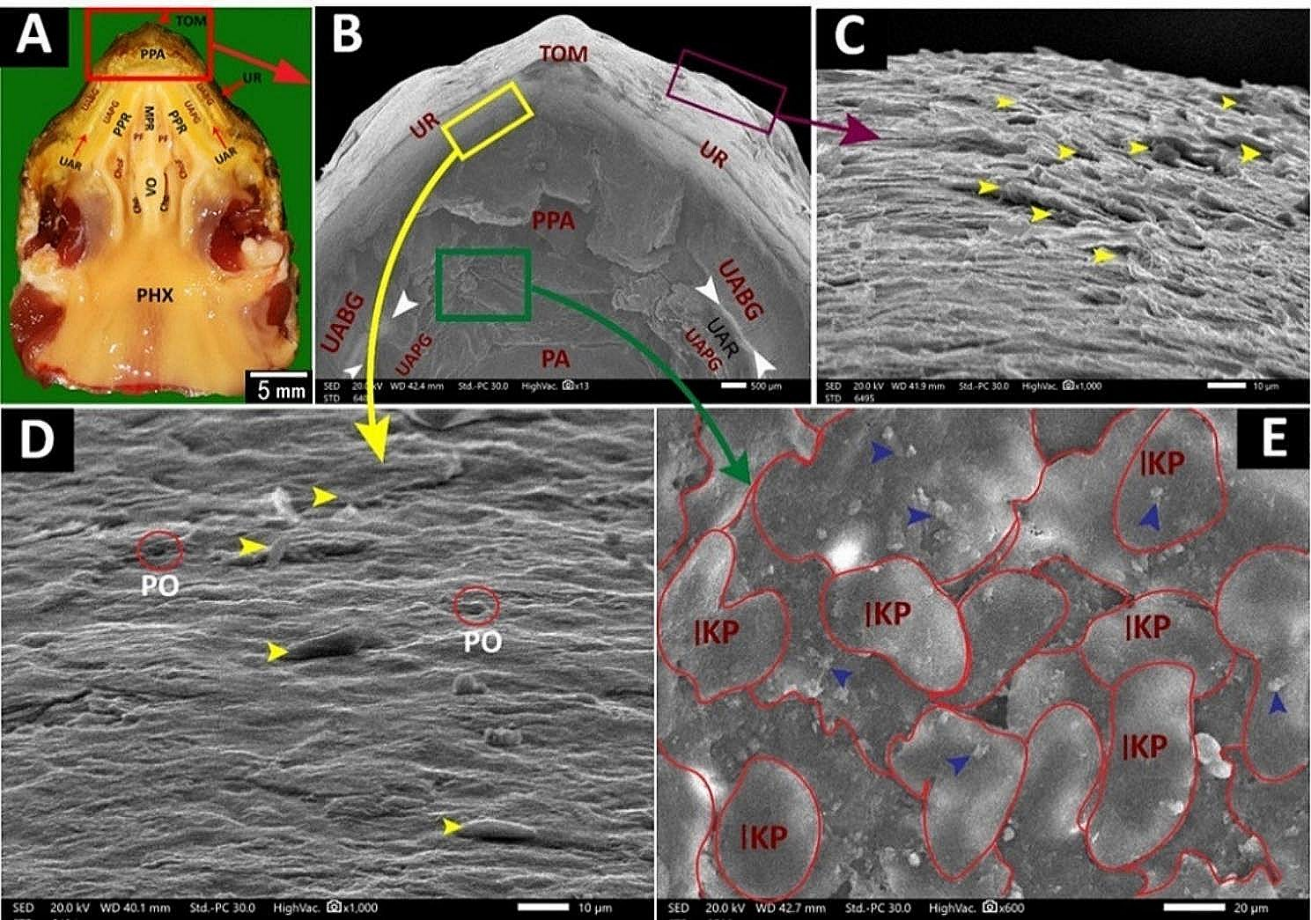
View (**A**) shows a gross anatomical image of the Greek tortoise oropharyngeal roof. Views (**B-E**) show SEM images of the most rostral part of its roof. Tomium (TOM), upper rhamphotheca (UR), peri palatine area (PPA), upper alveolo-beakal groove (UABG), upper alveolar ridge (UAR), upper alveolo-palatine groove (UAPG), peripheral palatine ridge (PPR), median palatine ridge (MPR), palatine area (PA), palatine fold (PF), vomer (Vo), choanae (Cho), choanal fold (ChoF), pharynx (PHX), pores (PO), interlocking keratinized epithelial plates (IKP) in the peri palatine area, Scales of keratinized epithelium directed rostro-medially (yellow arrowheads), upper alveolar ridge (white arrowheads), mucus secretions (blue arrowheads)

The **upper rhamphotheca** guarded the roof of the oropharyngeal cavity rostrally. It appeared as a large keratinized convex structure interrupted by tomiodonts (Fig. 6A, B, C, D).

**Fig. 7:**
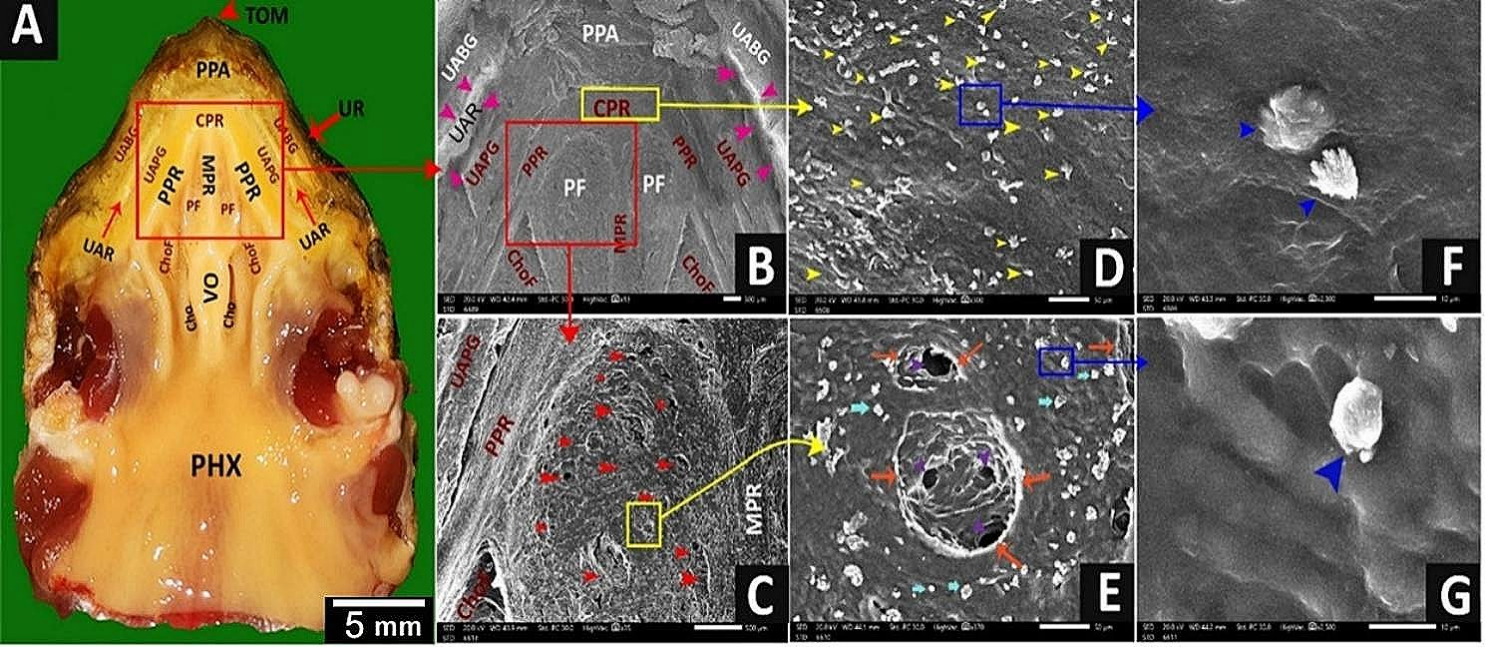
View (**A**) shows a gross anatomical image of the Greek tortoise oropharyngeal roof. Views (**B-G**) show SEM images of its peri choanal part. Tomium (TOM), upper rhamphotheca (UR), peri palatine area (PPA), upper alveolo-beakal groove (UABG), upper alveolar ridge (UAR), upper alveolo-palatine groove (UAPG), peripheral palatine ridge (PPR), median palatine ridge (MPR), vomer (Vo), choanae (Cho), choanal fold (ChoF), pharynx (PHX) core of palatine ridges (CPR), median palatine ridge (MPR) palatine fold (PF), the core of palatine ridge with scattered mucus secretions (Yellow arrowheads), mucus secretions (blue arrowheads), the palatine fold shows multiple salivary gland openings (red arrowheads), rim of the salivary gland (orange arrows) and pores of the gland (violet arrowheads)

**Fig. 8:**
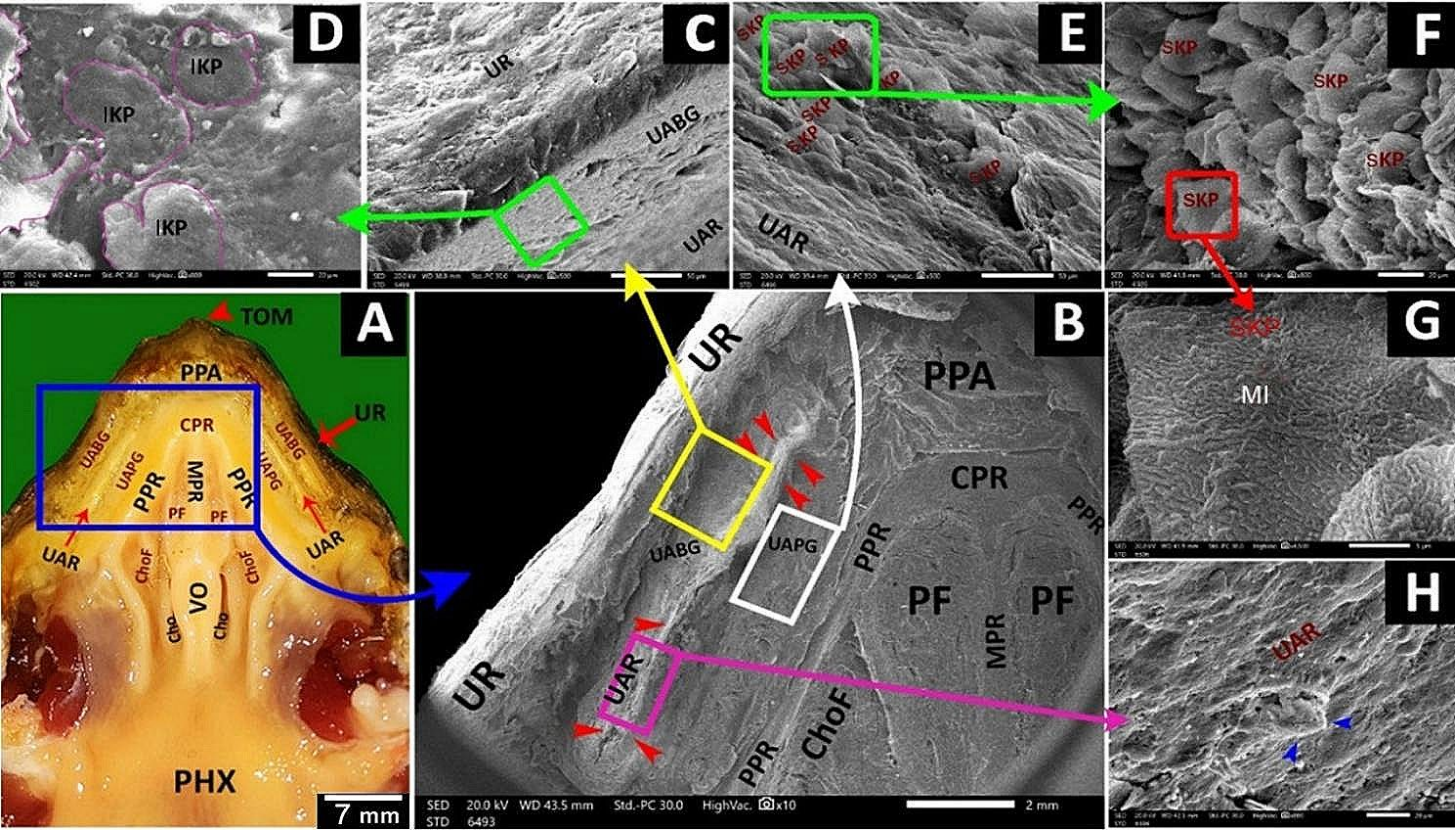
View (**A**) shows a gross anatomical image of the Greek tortoise oropharyngeal roof. Views (**B-H**) show SEM images of its lateral palatine area with different magnifications. Tomium (TOM), upper rhamphotheca (UR), peri palatine area (PPA) upper alveolo-beakal groove (UABG), upper alveolar ridge (UAR), upper alveolo-palatine groove (UAPG), peripheral palatine ridge (PPR), median palatine ridge (MPR), palatine fold (PF), vomer (Vo), choanae (Cho), choanal fold (ChoF), pharynx (PHX), core of palatine ridges (CPR), medial palatine ridge (MPR), interlocking keratinized epithelial plates (IKP), keratinized plates arranged as scales (SKP), microplicae (MI), keratinized projections (blue arrowheads)

The **peri-palatine region** was U-shaped, encircled by the palate rostro-laterally, and it was bordered laterally by the upper rhamphotheca and medially by a peripheral palatine ridge (Figs. 6B and 7A, B, D and F). The rostral part of the peri-palatine region and the upper alveolo-beakal groove had interlocking keratinized plates between them (Figs. 6A, B and E and 7A, B, C and F). The upper alveolo-palatine groove appeared rougher due to many keratinized plates arranged as fish scales (Figs. 6C and D and 8B, E, F and G). Each keratinized plate had surface microplicae (Fig. 8B, C, D, F, G). The upper alveolar ridge appeared rough, wavy, and serrated with keratinized projections (Figs. 7A and B and 8A, B and H).

**Fig. 9:**
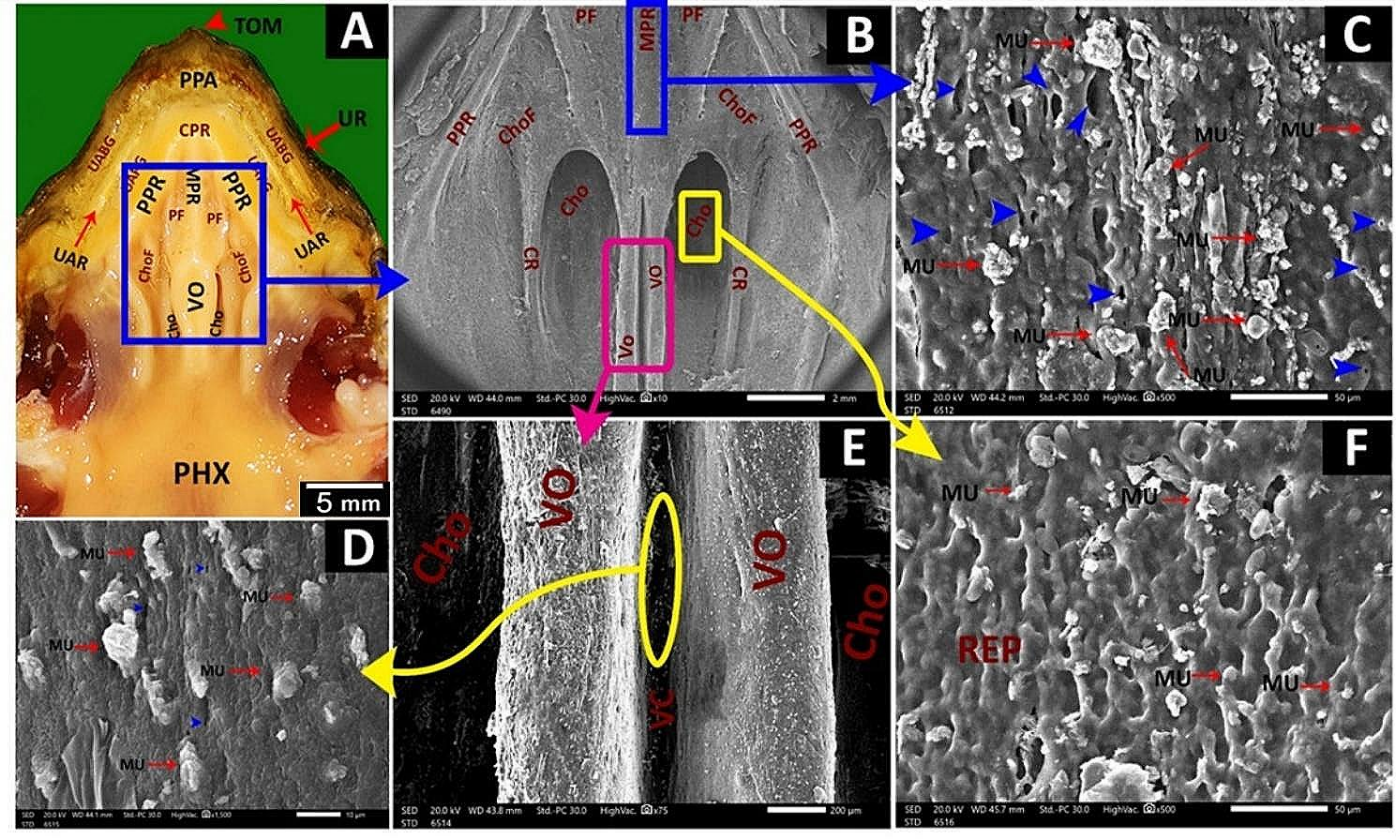
View (**A**) shows a gross anatomical image of the Greek tortoise oropharyngeal roof. Views (**B-F**) show SEM images of the middle palatine area with different magnifications. Tomium (TOM), upper rhamphotheca (UR), peri-palatine area (PPA), upper alveolo-beak groove (UABG), upper alveolar ridge (UAR), upper alveoli-palatine groove (UAPG), peripheral palatine ridge (PPR), median palatine ridge (MPR), palatine fold (PF), vomer (Vo), choanae (Cho), choanal fold (ChoF), pharynx (PHX), the core of palatine ridges (CPR), choanal ridge (CR), mucus secretions (MU), palatine glands opening (blue arrowheads), respiratory epithelium with mucus secretions (REP)

The **palato-choanal region**; the core of palatine ridges, palatine ridges, and vomer cleft had mucous glands and numerous glandular mucus secretions with different shapes (Figs. 7D and F and 9A, B, E, C and D). The palatine fold showed single and multi-porous palatine glands with obvious mucus secretions (Fig. 7C, E, G). The choanae appeared as two pine nut-shaped openings lined by respiratory epithelium with mucus secretions (Fig. 9B, F).

**Fig. 10:**
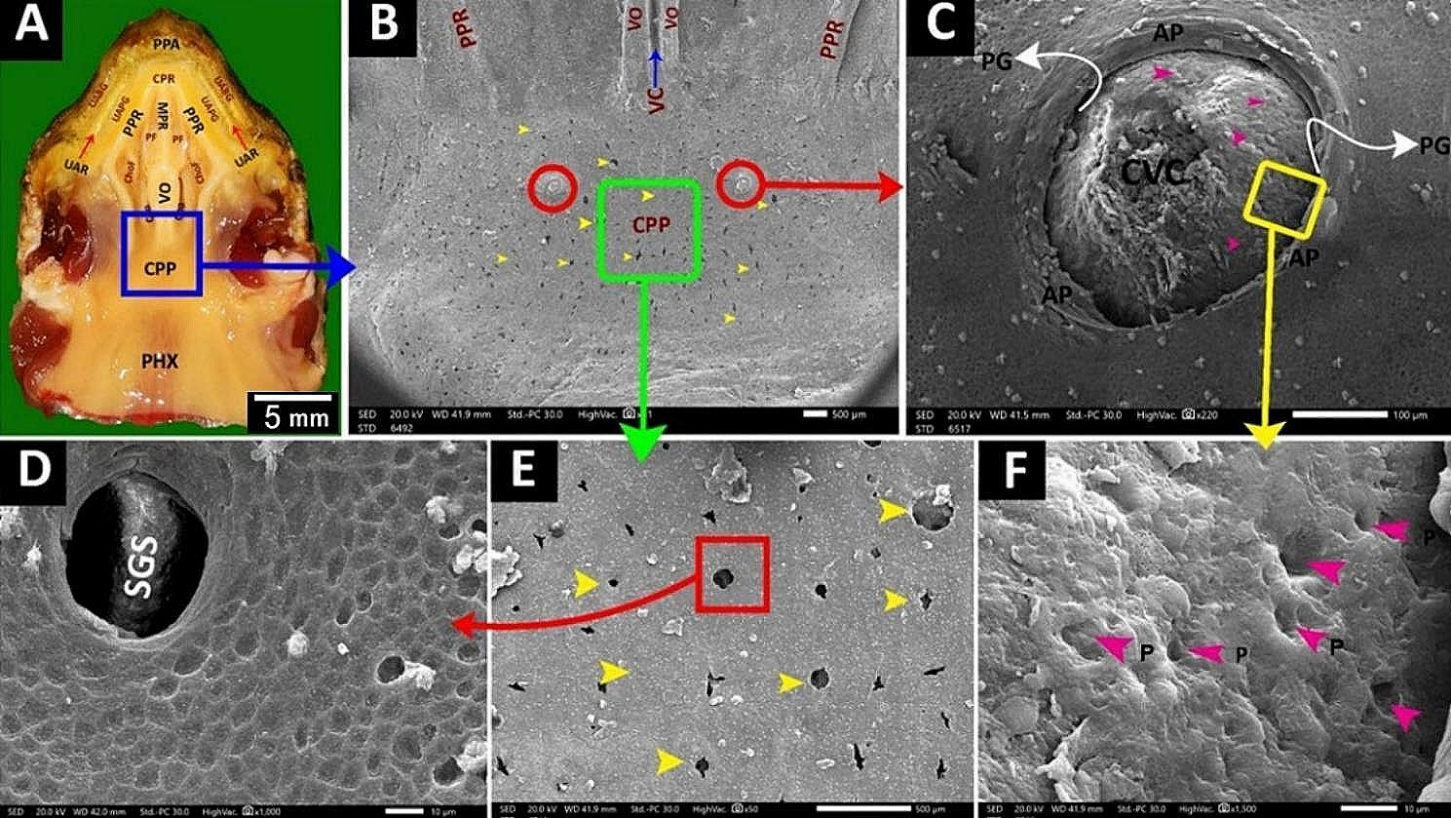
View (**A**) shows a gross anatomical image of the Greek tortoise oropharyngeal cavity roof. Views (**B-F**) show SEM images of the caudal palatine area with different magnifications. Upper rhamphotheca (UR), peri palatine area (PPA), upper alveolo-beakal groove (UABG), upper alveolar ridge (UAR), upper alveolo-palatine groove (UAPG), peripheral palatine ridge (PPR), median palatine ridge (MPR), palatine fold (PF), vomer (Vo), choanae (Cho), choanal fold (ChoF), pharynx (PHX), the core of palatine ridges (CPR), vomer cleft (VC), caudal palatine part (CPP), circumvallate like papilla with vallate core (CVC), papillary groove (PG), annular pad (AP), pores (P), openings of salivary glands (yellow arrowheads), salivary gland secretion (SGS)

The **caudal palatine region** was situated between the choanae’s end and the pharynx’s beginning (Fig. 10A, B). With higher magnification, there was a single pair of circumvallate-like papilla with multiple salivary gland openings and secretions (Fig. 10B, C, E, F). The circumvallate-like papilla carried pores (Fig. 10C, F).

### SEM characterizations of the oropharyngeal roof of red-eared slider

**Fig. 11:**
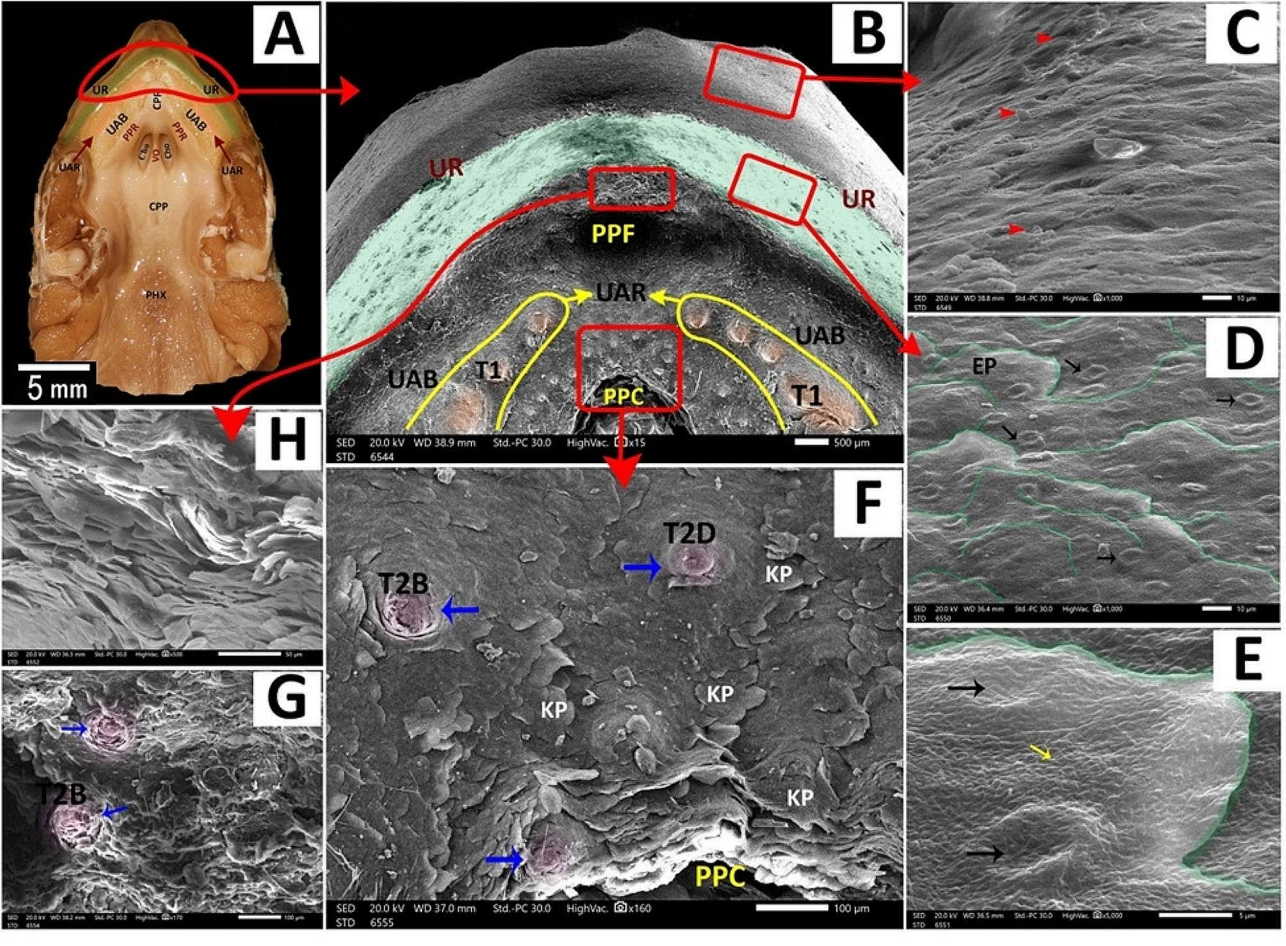
View (**A**) shows a gross anatomical image of the red-eared slider oropharyngeal cavity roof. Views (**B-H**) show SEM images of the rostral part of the roof with different magnifications. Upper rhamphotheca (UR), the core of palatine ridge (CPR), upper alveolar band (UAB), upper alveolar ridge (UAR), Peripheral palatine ridge (PPR), Choanae (Cho), vomer (VO), caudal palatine part (CPP) and pharynx (PHX), peri palatine fossa (PPF), type1 tooth-like projections (T1), peri palatine cleft (PPC). Keratinized surface epithelium (red arrowheads), the surface epithelium (EP), button-like structures (black arrows), surface microplicae (yellow arrows), keratinized epithelium plates (KP), Type 2 tooth-like projections (blue arrows), Type 2, Shape B tooth-like projection (T2B), type 2, and shape D tooth-like projections (T2D)

The **upper rhamphotheca** had layers of keratinized epithelium, button-like structures, and surface microplicae (Fig. 11A, B, C, D, E, H). A median peri-palatine fossa was on the upper rhamphotheca (Fig. 11B, H).

**Fig. 12:**
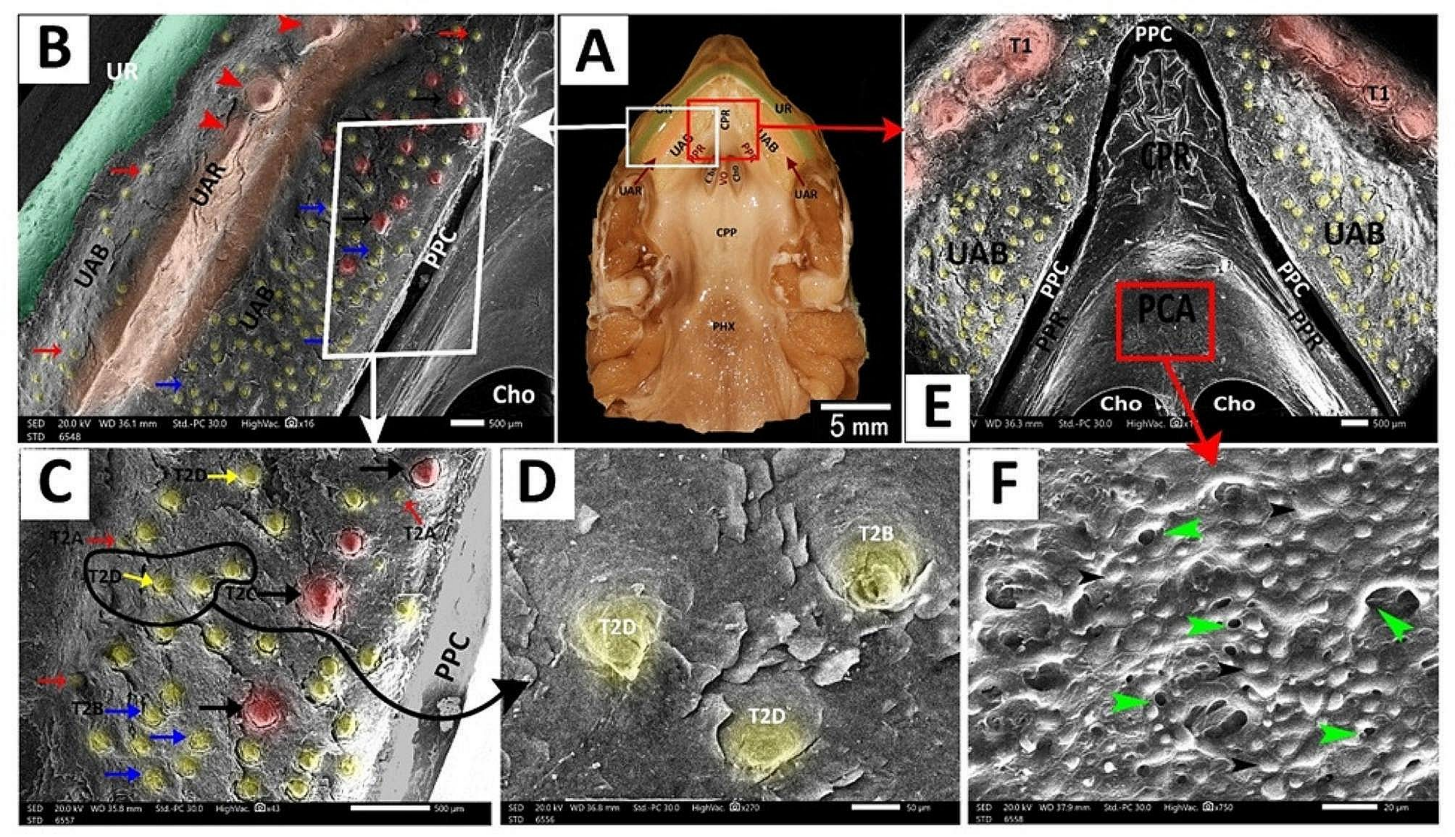
View (**A**) shows a gross anatomical image of the red-eared slider oropharyngeal roof. Views (**B-F**) show SEM images of the rostral palatine region of the roof with different magnifications. The core of palatine ridges (CPR), peripheral palatine ridge (PPR), Choanae (Cho), vomer (VO), caudal palatine part (CPP), pharynx (PHX), peri-palatine cleft (PPC), upper alveolar ridge (UAR) containing type1 tooth-like projections (red arrowheads) appeared as a large cone shape, the upper alveolar band (UAB) having different shapes of type 2 tooth-like projections; type 2 shape A (T2A) appears as pimple-like elevation (red arrows), type 2 shape B (T2B) appears as artichoke like elevation (blue arrows), type 3 shape C (T2C) appears as small cone shape surrounded by a groove (black arrows) and type 2 shape D (T2D) appears as small cone shape didn’t surrounded a groove (yellow arrows), peri-choanal area (PCA), palatine glands (green arrowheads)

The **upper alveolar band** was a large V-shaped structure surrounding the palate-choanal region. With higher magnification, it had keratinized epithelial plates, two upper alveolar ridges, one on each side, and scattered teeth-like projections (Figs. 11B, F and G and 12A, C, D and E). These projections appeared in two shapes: large cone-shaped projections arranged in a line to be the upper alveolar ridge (Figs. 11B and 12A and B) and scattered projections all over the upper alveolar band that appeared in four different projections; pimple-like projections, artichoke-like projections, small cone-shaped projections with surrounding groove and small cone-shaped projections without surrounding groove (Figs. 11B, F and G and 12B and E).

The **palato-choanal region** was separated from the upper alveolar band by a small peri-palatine cleft (Fig. 12E). This region started with a large cone-shaped core of palatine ridges (Fig. 12E). The peripheral palatine ridges were two ridges that originated from the core of palatine ridges and extended till the level of the choanal end (Fig. 12E A, B).

**Fig. 13:**
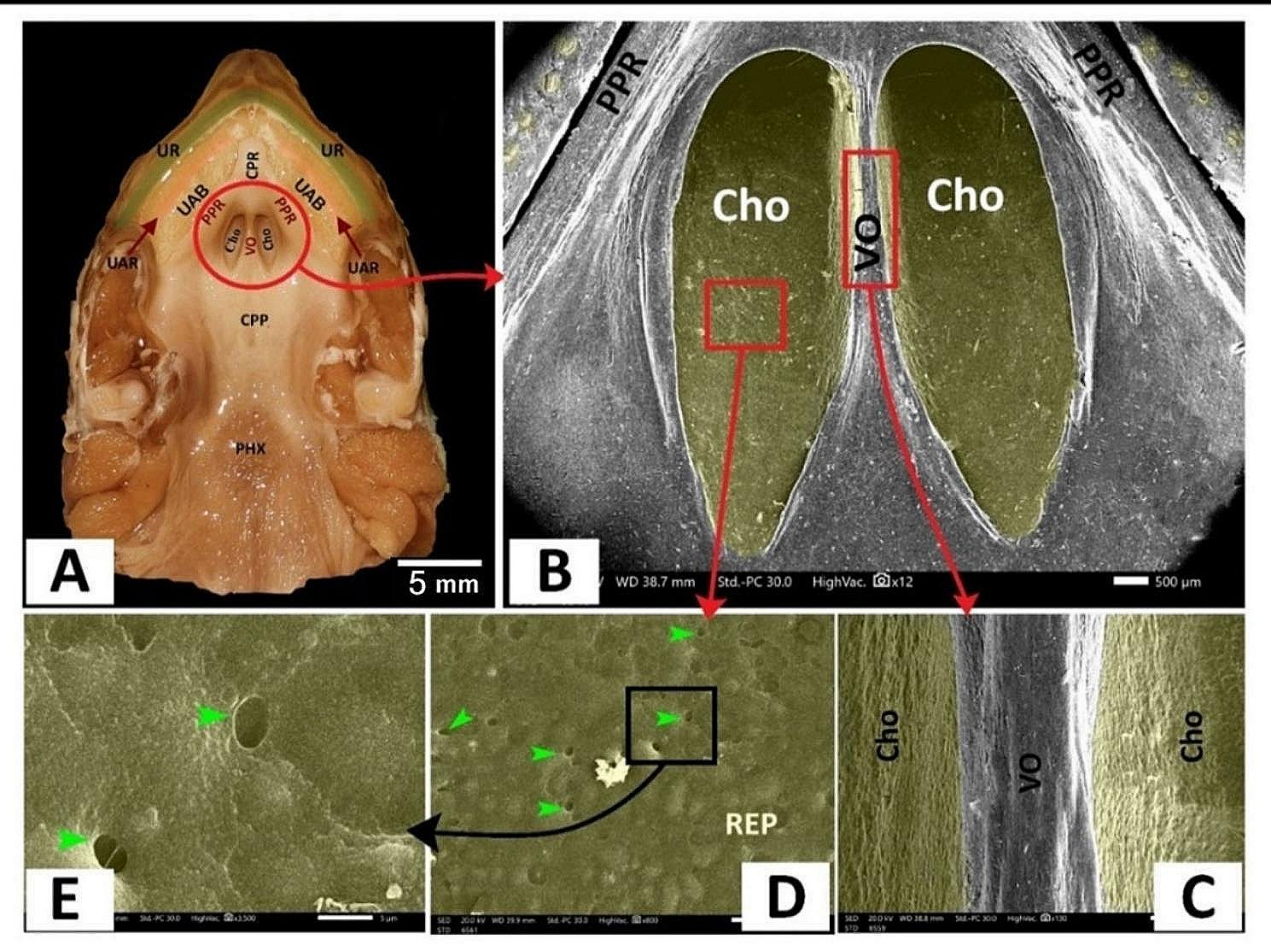
View (**A**) shows a gross anatomical image of the red-eared slider oropharyngeal cavity roof. Views (**B-E**) show SEM images of the choanal region with different magnifications. Upper rhamphotheca (UR), core of palatine ridge (CPR), upper alveolar band (UAB), upper alveolar ridge (UAR), Peripheral palatine ridge (PPR), Choanae (Cho), vomer (VO), caudal palatine part (CPP) and pharynx (PHX), choanal glands openings (green arrowheads)

The peri-choanal region was the most cranial part of the palate rostral to the choanae. With higher magnification, it showed palatine glands (Fig. 12E, F). The choanae are two elliptical openings separated by vomer and lined with respiratory epithelium with choanal gland openings (Fig. 13B, D, E). The vomer appeared as a short, straight crest between the two choanal openings (Fig. 13B, C).

**Fig. 14:**
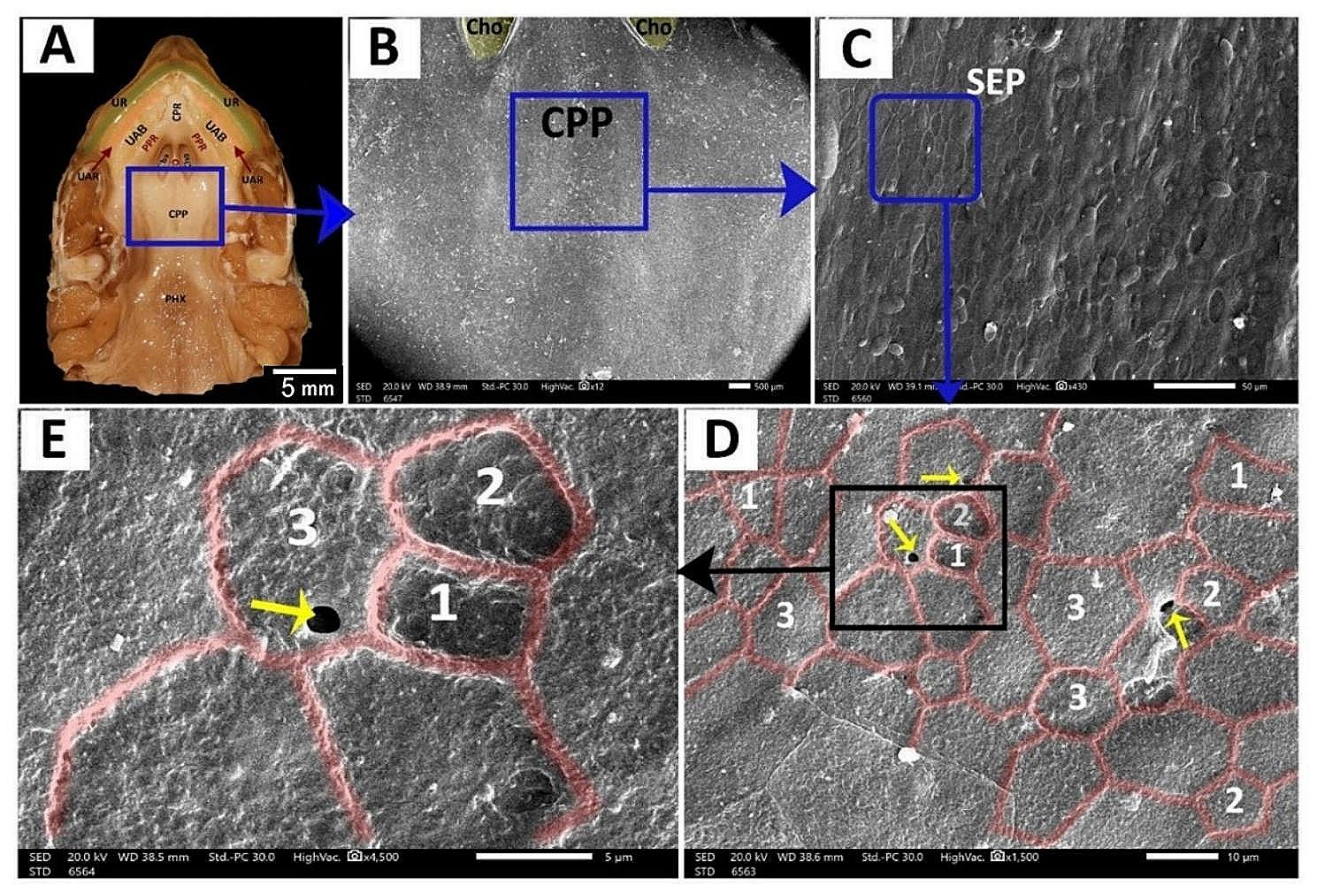
View (**A**) shows a gross anatomical image of the red-eared slider oropharyngeal cavity roof. Views (**B-E**) show SEM images of the caudal palatine region with different magnifications. Upper rhamphotheca (UR), core of palatine ridge (CPR), upper alveolar band (UAB), upper alveolar ridge (UAR), Peripheral palatine ridge (PPR), Choanae (Cho), vomer (VO), caudal palatine part (CPP), pharynx (PHX), surface epithelium (SEP), palatine glands (yellow arrows) with poorly keratinized epithelium geometrically arranged into four-sided shape (1), five-sided shape (2) and six-sided shape (3)

The **caudal palatine region** was situated between the choanae’s end and the pharynx’s beginning (Fig. 14A, B). With higher magnification, its epithelium appeared poorly keratinized and geometrically arranged into four-sided, five-sided, and six-sided patterns with few palatine glands’ openings (Fig. 14C, D, E).

### LM characterizations of the oropharyngeal roof of Greek tortoise

**Fig. 15:**
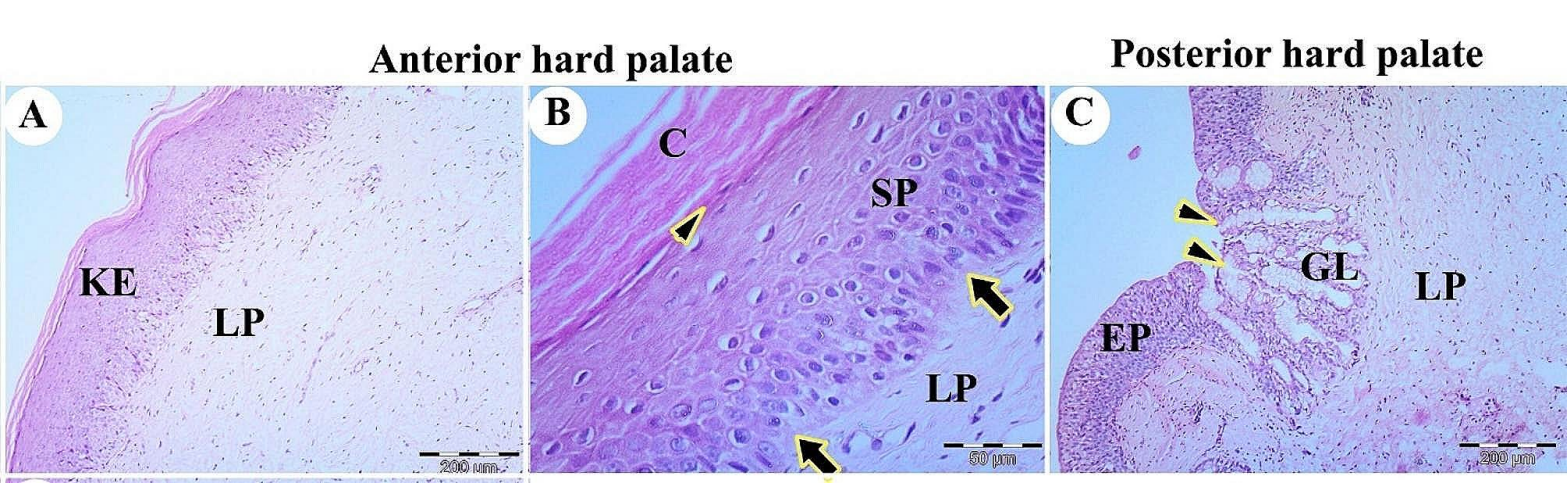
A histological micrograph shows the dorsal surface of the palate of the Greek tortoise. Views (**A** & **B**) reveal the keratinized stratified epithelium of mucosal surface (KE), lamina propria (LP), stratum basalis on the folded basement membrane (arrows), stratum spinosum (SP), stratum superficial (arrowhead), and stratum corneum (**C**). View (**C**) shows the low keratinized epithelium (EP), lamina propria (LP), mucous gland (GL), and gland orifices through the surface epithelium (arrowheads). H&E, Mag. 100X, 400X, 100X, bars = 200, 50, 200 μm, respectively

**Fig. 16:**
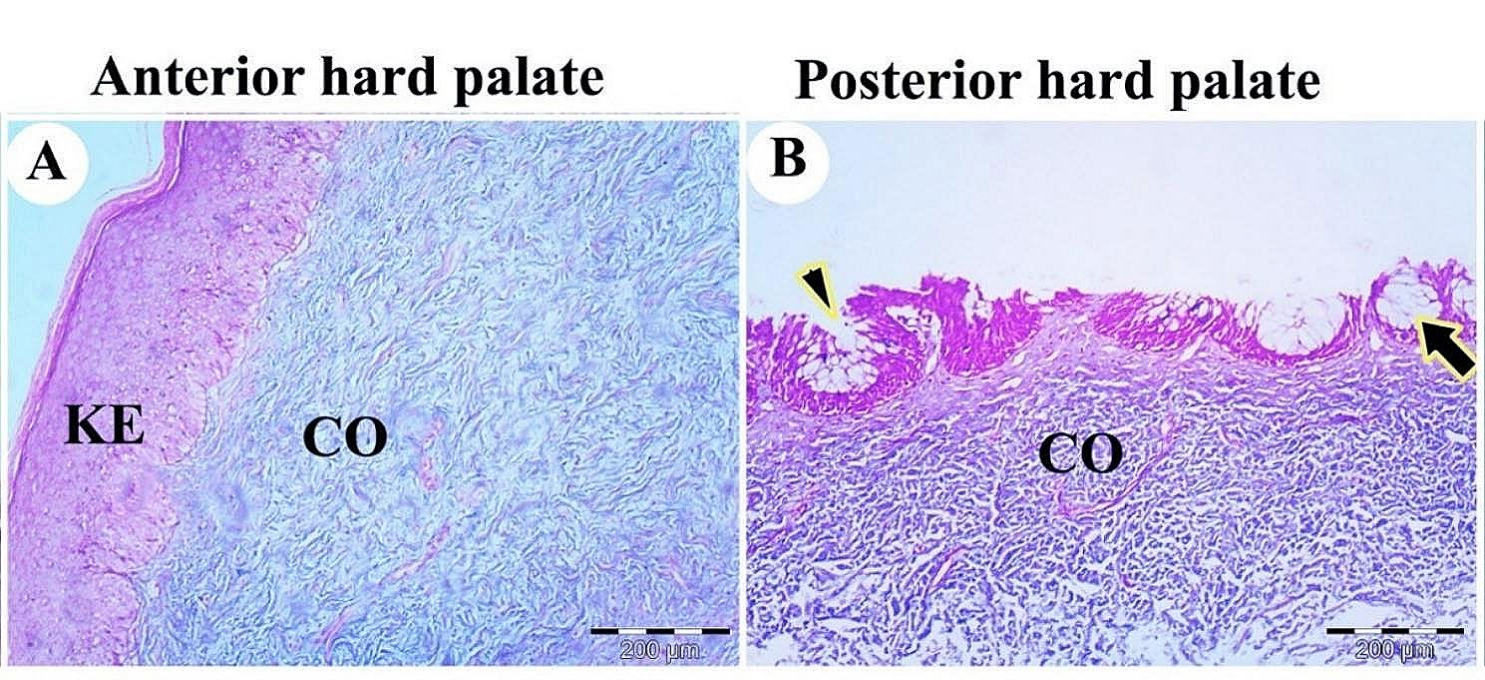
A histological micrograph shows the dorsal surface of the palate of the Greek tortoise. View (**A**) at the anterior part of the palate reveals keratinized stratified epithelium of mucosal surface (KE), lamina propria with collagen fibers (CO); View (**B**) at the caudal part of the palate shows low keratinized epithelium, lamina propria with collagen fiber (CO), mucous gland (black arrow) and gland orifices through the surface epithelium (black arrowhead). Masson’s trichome, Mag. 100X, 100X. bars = 200 μm, 200 μm, respectively

The rostral part of the palate had stratified squamous epithelium with a thick keratin layer. The epithelium consisted of a thick keratinized layer (stratum corneum), stratum superficial, stratum spinosum, and stratum basalis. The stratum basalis was a columnar epithelium with basophilic cytoplasm arranged at a corrugated and folded basement membrane. The lamina propria of dense irregular connective tissue was present beneath the palate mucosal surface epithelium (Figs. 15A, B and C and 16A).

The caudal part of the palate had less keratinized epithelium and mucous gland acini closely attached to the surface epithelium with specific architecture. The mucous gland had orifices through the epithelium. The lamina propria consisted mainly of collagen fibers (Fig. 16B).

**Fig. 17:**
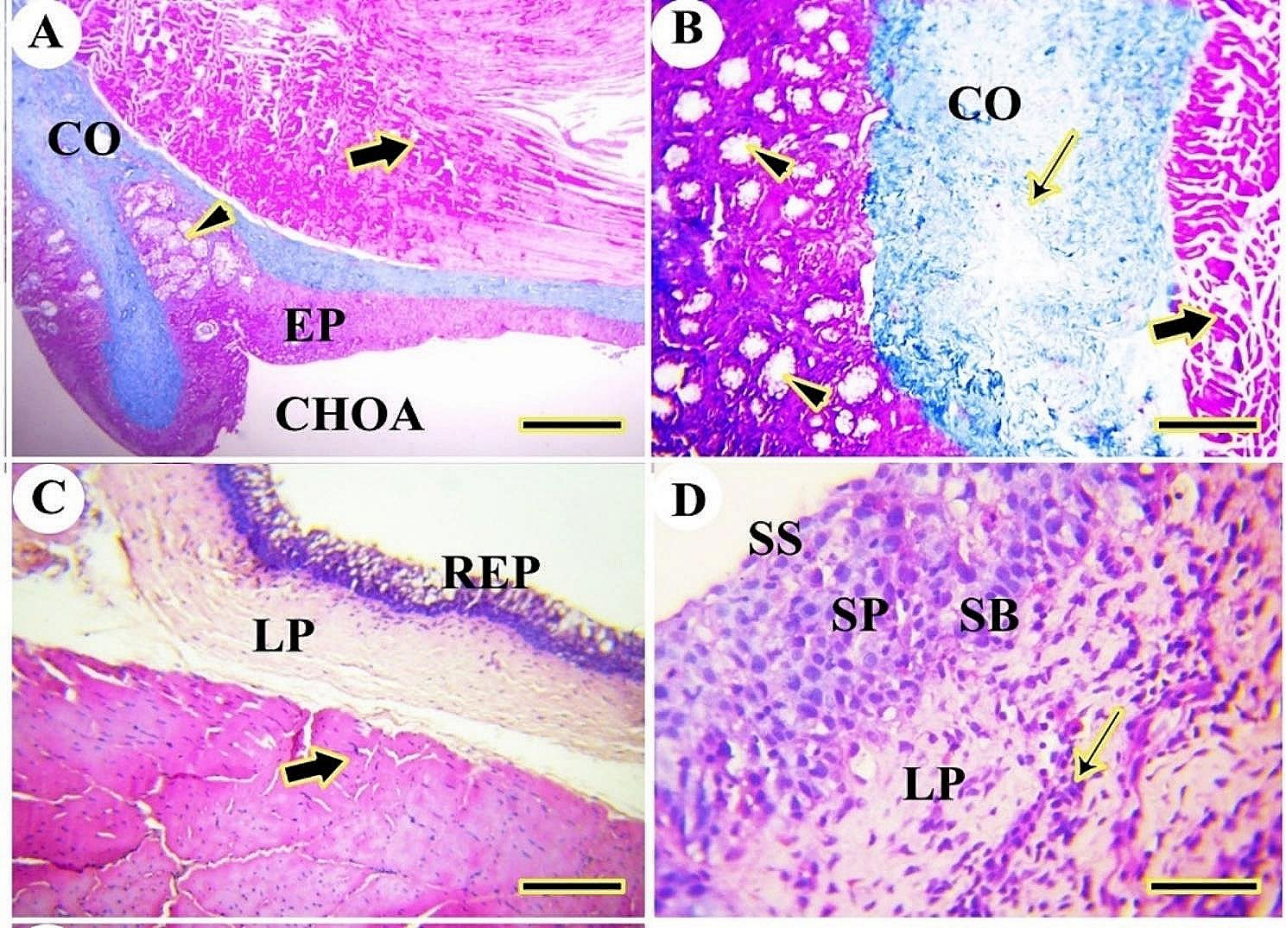
A histological micrograph shows the choanae of the Greek tortoise in views (**A**&**B**), Choanal opening (CHOA), the stratified epithelium (EP), mucous gland (arrowhead), collagen fiber (CO), and muscle (thick arrow). (Masson trichrome, Mag.100X, and 400X, bar = 500, 200 μm respectively). View (**C**) shows the respiratory epithelium (REP), lamina propria (LP), and skeletal muscle (thick arrow). (H&E. Mag. 100X, bar = 100 μm). View (**D**) shows a higher magnification of stratified squamous epithelium showing stratum superficial (SS), stratum spinosum (SP), stratum spinosum (SB), lamina propria (LP), and diffuse lymphatic tissue (thin arrow) (H&E. Mag.400X, bar = 50 μm)

Near the oral cavity, the choanae were lined by stratified squamous epithelium, but they were lined by respiratory epithelium with goblet cells near the nasal cavity. The stratified epithelium consisted of a superficial layer of squamous epithelium, stratum spinosum, and stratum basalis lining the basement membrane. The lamina propria and skeletal muscles were observed beneath the mucosal epithelium. The lamina propria had mucous glands acini and collagen fibers (Fig. 17A, B, C, D).

**Fig. 18:**
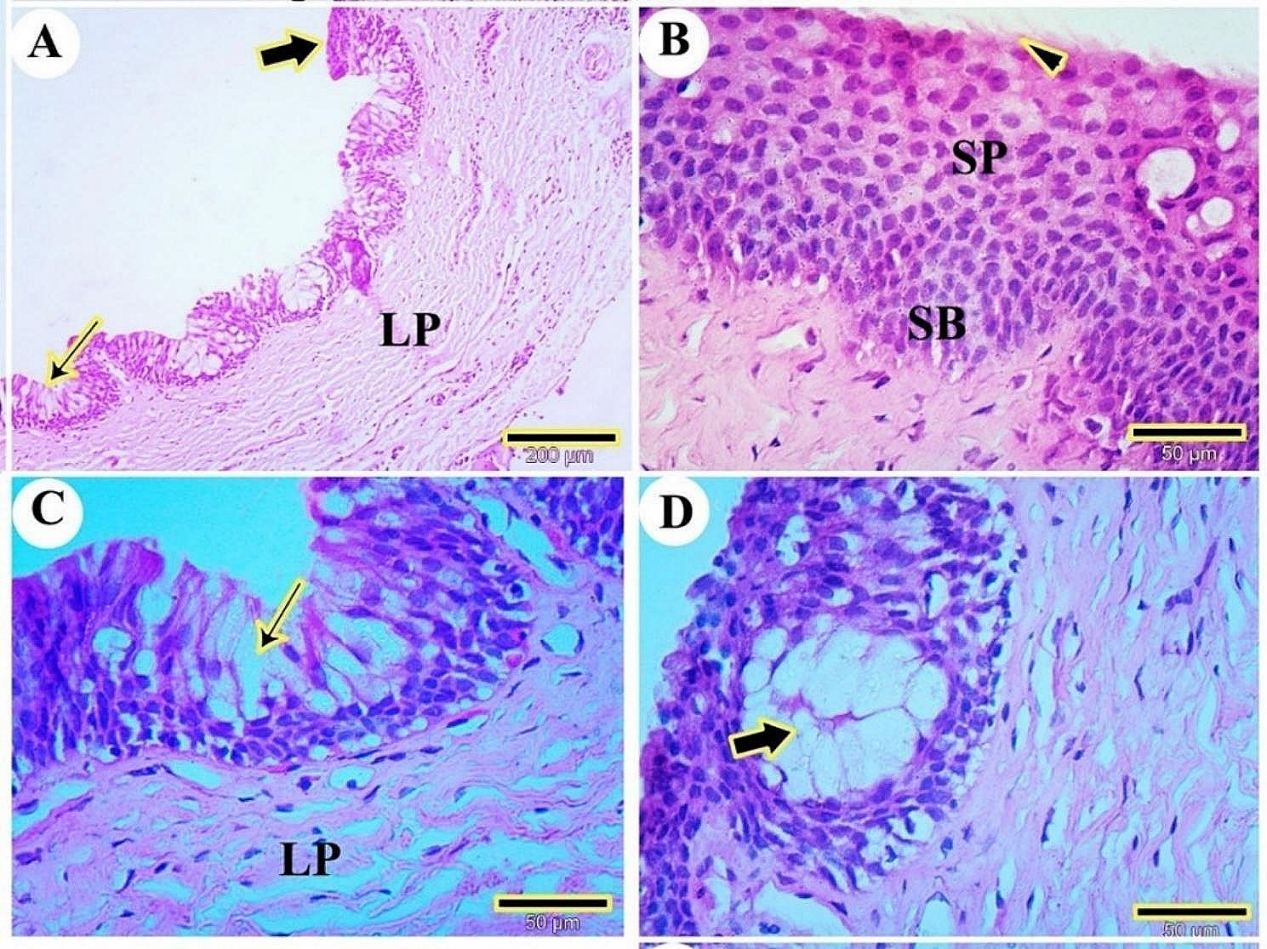
A histological micrograph shows the palate of the red-eared slider. View (**A**) shows the stratified epithelium of the anterior part and the transformation of the epithelium of the caudal part into pseudostratified with a high number of mucous cells and lamina propria (LP) (H&E, Mag.100X, bar = 200 µ). View (**B**) shows stratum basalis (SB), stratum spinosum (SP), and stratum superficial (arrowhead) (H&E, Mag.400X, bar = 500 µ). Views (**C**&**D**) show mucous (thin arrow), lamina propria (LP), and mucous gland (thick arrow) (H&E, Mag.400X, bar = 50 µ)

### LM characterizations of the oropharyngeal roof of the red-eared slider

The mucosal surface of the rostral part of the palate consisted of slightly keratinized stratified squamous epithelium arranged as a thin cornified layer, stratum superficial, stratum spinosum, and stratum basalis set on the folded basement membrane. The lamina propria of the palate was located beneath the epithelium and consisted mainly of collagen fibers. The epithelium of the caudal part of the palate had a lot of mucous cells and mucous glands acini (Fig. 18A, B, C, D).

**Fig. 19:**
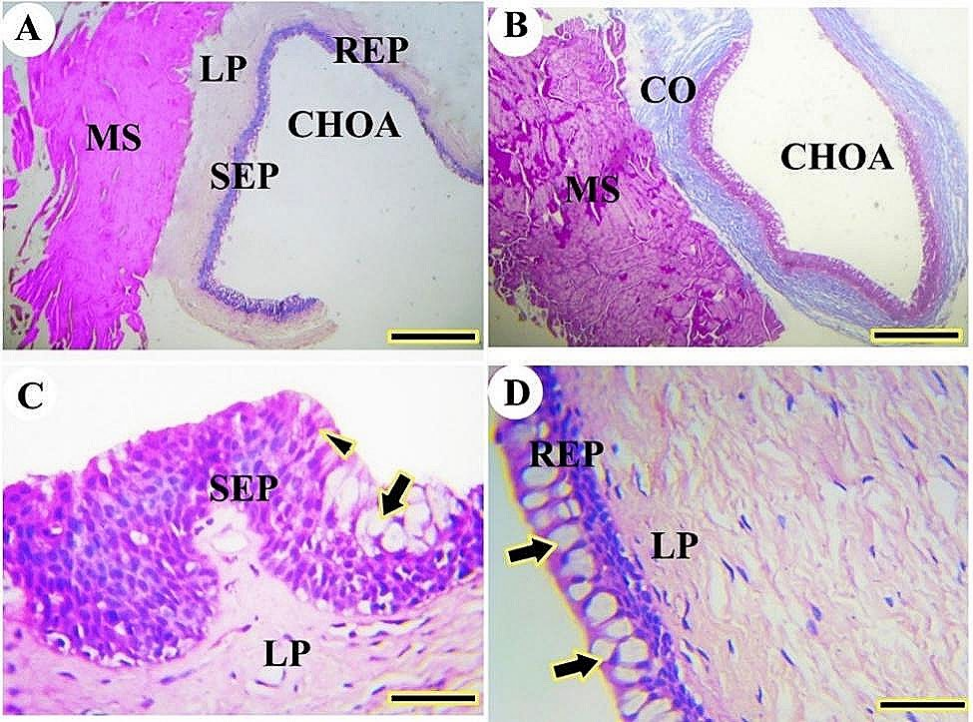
A histological micrograph shows the choanae of the red-eared slider. Views (**A** & **B**) show the choanal opening (CHOA), level of respiratory epithelium (REP), level of stratified epithelium (SEP), lamina propria (LP), skeletal muscle (MS) and collagen fibers (CO). (H&E, Masson trichrome respectively, Mag.400X, bar = 500 µ). View (**C**) shows the stratified epithelium of olfactory mucosa (SEP), olfactory cells (arrowhead), goblet cells (arrow), and lamina propria (LP). (H&E, Mag.400X, bar = 50 µ). View (**D**) shows the respiratory epithelium of choanae (REP), goblet cells (arrows), and lamina propria (LP) (H&E, Mag.400X, bar = 50 µ)

The choanae were lined by sensory stratified epithelium and respiratory epithelium. The lamina propria and striated muscles were located beneath the mucosal epithelium. The lamina propria had collagen fibers (Fig. 19A, B). The sensory epithelium of choanae had superficial olfactory cells (Fig. 19C). The respiratory epithelium was a pseudostratified columnar epithelium with many goblet cells (Fig. 19D).

## Discussion

The turtles were roughly generalized or specialized in their food habits and diet [29]. The aquatic and semiaquatic turtles were good examples of physical and behavioral diversities and their adaptability to the feeding medium and food type; most of them adapted to both land and water feeding [15]. The majority of chelonians could exhibit dietary fluctuations during their lifespan and may transform from carnivorous to omnivorous and herbivorous behaviors [30]. Meanwhile, the Greek tortoises specialized in their feeding habits; it is a strictly herbivorous chelonians feeding on grasses, weeds, leafy greens, flowers, and occasional fruits [31].

The turtle anatomy may have an analogous mechanism for both food gathering and defense, similar to birds [32]. The upper rhamphotheca of turtles is a prime example of an adaptation to feeding ecology and dietary behavior that has a compensatory role for teeth as long as turtles are toothless creatures [33], avian are also beaked edentulous species [34]. Tomiodonts are not real teeth, but they function as incisor teeth in mammals, biting and crushing food with the help of the serrated lateral edge [33].

The current study is the first to focus on the peri-palatine area, which appeared as a rough band resembling the upper rhamphotheca. This area included the upper alveolar ridge, which appeared wavy and had pointed projections that faced the lower rhamphotheca. All of these structures appeared to act as a crushing surface inside the mouth, compensating for the lack of teeth. The jaws’ crushing surfaces were located in the maxillary dentary and the mandible’s alveolar surface, which varied in width and ridges [33]. The omnivorous red-eared slider, like Blanding’s turtle (*Emydoidea blandingii*), Spotted turtles (*Clemmys guttata*), and *Trachemys* species, has a sharp cutting edge of the upper rhamphotheca and a median premaxillary notch, allowing it to bite powerfully despite lacking teeth [33]. Meanwhile, the omnivorous estuarine river turtle (*Batagur baska*) has a deep premaxillary notch with bicuspid Tomiodonts [35]. The beak is the only substitute for teeth in edentulous turtles [36].

The choanal opening is more significant and wider in the red-eared slider compared to that of the Greek tortoise, which appeared narrow and slightly away from the upper rhamphotheca, whereas the small size of choanae in terrestrial tortoises was due to the complexity of the palate that occupied by ridges and folds [19]. The current investigation of the numerous ridges and folds at the palatine region in Greek tortoises is supposed to increase the surface area of the palatal mucosa with some roughness to share in food processing, the function of the palate to separate the oral and nasal cavity, it had a role in food processing [37] and numerous palatine ridges in land tortoise species as the *Testudo hermanni*; these ridges played a function in keeping the first bite of food when the tongue is stretched out of the mouth to capture a second piece of food [17]. Within the same fact, the two choanal folds observed in the current study of Greek tortoises have another essential function to minimize the size of the choanal opening, especially when eating grasses in a dusty environment [19].

The red-eared slider oropharyngeal roof appeared more straightforward than the roof of other tortoises; this simplicity in the oropharyngeal roof structures was also seen in semiaquatic Malayan box turtles (*Cuora amboinensis*) [38]. Moreover, the turtles that tended to be aquatic showed more oropharyngeal simplicity than other land tortoises [17]. The upper alveolar band consisted of keratinized epithelial plates with two upper alveolar ridges, one on each side, and scattered teeth-like projections, indicating that chewing took place primarily in the rostral palate and the area near the upper and lower rhamphotheca due to roughness and keratinization [39].

SEM analysis of the peri-palatine region in the Greek tortoise revealed a highly keratinized area with multiple interlocking keratinized plates, particularly at the rostral part, and it has a serrated upper alveolar ridge with a compensatory role of the tooth, where keratinization is commonly associated with terrestrial feeding [40]. In the red-eared slider, the tooth-like projections at the peri-palatine region had not been thoroughly studied or described except for the description of tomiodonts and serration of the upper rhamphotheca and the roughness of the rostral palate [33].

The palato-choanal region in Greek tortoises is a common feature for terrestrial feeding; ridges share in the chewing process and food friction while mucus secretion keeps moistening and lubricating the oral mucosa and facilitates food movement [38]. The red-eared slider palatine ridges were devoid of mucus glands, unlike other terrestrial tortoises including the Greek tortoise, also in Malayan box turtle (*Cuora amboinensis*) [38], and Giant Asian pond turtle (*Heosemys grandis)* [39].

The current study is the first to reveal the presence of palatine papillae in tortoises, while all the previous studies worked on the different shapes of lingual papillae among turtles and tortoises [41]. On the other hand, different shapes of palatine papillae were commonly observed in avian species such, as Europea Magpie (*Pica pica*) and Common Raven (*Corvus corax*) [42]. The caudal palatine region in the red-eared slider *and Trachemys venusta* showed poorly keratinized epithelium, geometrically arranged into four-sided, five-sided, and six-sided patterns with few salivary gland openings [43].

Histologically, the Greek tortoise oral cavity was lined by stratified squamous epithelium that was commonly reported in testudines. Meanwhile, the oral cavity is lined by stratified cuboidal epithelium in freshwater turtle (*Geoclemys reevesii)* [44], Chinese soft-shelled turtle (*Pelodiscus sinensis*) [45], Asian snail-eating turtle (*Malayemys subtrijuga*) [46], and Pacific ridley turtle (*Lepidochelys olivacea*) [47]. The oral cavity’s lining epithelium was more keratinized in the Greek tortoise than the red-eared slider. The thick keratin layer covering the stratified squamous epithelium was previously recorded in a wide range of marine turtles and terrestrial tortoise such as hawksbill turtle (*Eretmochelys imbricata bissa*) [48], basal tortoise (*Manouria emys emys*) [20] and Egyptian tortoise (*Testudo kleinmanni*) [49], while the keratinization was less observed or even absent in the epithelium of freshwater turtles [41]. Moreover, keratinization protects the oral mucosa from abrasions and dehydration in dry environment challenges. Additionally, the degree of keratinization of the oral mucosa is associated with food friction and processing [50]. *Trachemys* have up to 16 species, with six being polytypic. *Trachemys* taxonomic characteristics, such as coloration and markings, were sometimes described from nonliving specimens [43]. Keratinization of the lingual epithelium was thought to have occurred concurrently with the evolution of amniotes [51]. We propose including the degree of keratinization in the oral cavity as a taxonomy character. The mucous cells are found at the entire epithelial surface of the oral cavity, the role of keratinization is to tolerate dehydration and lubricate the oral mucosa [52]. This is an excellent example of the ecological adaption of land tortoise feeding for more lubrication and moistening of dry food besides helping for food capture in Testudines species [53]. The Greek tortoises have highly keratinized epithelium beside the well-developed musculature of the tongue that had a role in the adaptation of feeding habits and terrestrial lifestyle [54]. The oral glands were utterly absent in the peri-choanal palate of the basal tortoise (*Manouria emys emys*) [20]. Moreover, the palatine keratinization and salivary gland distribution were previously described in the semiaquatic giant Asian pond turtle (*Heosemys grandis*) [39]. The palatine mucosa had a more sensory role than the secretory role due to taste buds in the surface epithelium, and the lamina propria had nerve endings [38]. The taste buds have a chemosensory function [55].

The choanae of the Greek tortoise and red-eared slider have two openings lined by sensory pseudostratified respiratory epithelium, like in *T. adiutrix* choanae [41]. The choanae serve as air passages connecting the oral and nasal cavities. The ellipsoid and elliptical shapes are familiar to testudines choanae [53].

The pharyngeal region in both species was lined by stratified epithelium with a thin keratin layer. Still, the lamina propria in the Greek tortoise showed multiple mucus glands with surface openings to help the bolus slide easily into the esophagus. In the red-eared slider, the mucus glands were fewer as they depended on suction feeding [50].

## Conclusion

Considering that the red-eared slider *(Trachemys scripta*) is an omnivorous reptile, and the Greek tortoise (*Testudo graeca*) is an herbivorous one, the present work revealed their oropharyngeal roof adaptation to their feeding behavior. The red-eared slider upper rhamphotheca was sharper beside a spiky, pointed, long upper alveolar ridge and two teeth-like projections at its peri-palatine area. Greek tortoises had numerous ridges and folds in the palatine region and small choanal openings with two choanal folds.

## Data Availability

The datasets used and/or analyzed during the current study are available from the corresponding author on reasonable request.
